# Pattern of TAAR5 Expression in the Human Brain Based on Transcriptome Datasets Analysis

**DOI:** 10.3390/ijms22168802

**Published:** 2021-08-16

**Authors:** Anastasia N. Vaganova, Ramilya Z. Murtazina, Taisiia S. Shemyakova, Andrey D. Prjibelski, Nataliia V. Katolikova, Raul R. Gainetdinov

**Affiliations:** 1 Institute of Translational Biomedicine, St. Petersburg State University, Universitetskaya nab. 7/9, 199034 St. Petersburg, Russia; anastasia.n.vaganova@gmail.com (A.N.V.); ramilya.murtazina@gmail.com (R.Z.M.); taisia070296@mail.ru (T.S.S.); andrewprzh@gmail.com (A.D.P.); nkatolikova@gmail.com (N.V.K.); 2 St. Petersburg University Hospital, St. Petersburg State University, Universitetskaya nab. 7/9, 199034 St. Petersburg, Russia

**Keywords:** trace amines, trace amine-associated receptor, TAAR, TAAR5, human brain, brain cortex, striatum, white matter, major depressive disorder, HIV-encephalitis, Down syndrome

## Abstract

Trace amine-associated receptors (TAAR) recognize organic compounds, including primary, secondary, and tertiary amines. The TAAR5 receptor is known to be involved in the olfactory sensing of innate socially relevant odors encoded by volatile amines. However, emerging data point to the involvement of TAAR5 in brain functions, particularly in the emotional behaviors mediated by the limbic system which suggests its potential contribution to the pathogenesis of neuropsychiatric diseases. TAAR5 expression was explored in datasets available in the Gene Expression Omnibus, Allen Brain Atlas, and Human Protein Atlas databases. Transcriptomic data demonstrate ubiquitous low TAAR5 expression in the cortical and limbic brain areas, the amygdala and the hippocampus, the nucleus accumbens, the thalamus, the hypothalamus, the basal ganglia, the cerebellum, the substantia nigra, and the white matter. Altered TAAR5 expression is identified in Down syndrome, major depressive disorder, or HIV-associated encephalitis. Taken together, these data indicate that TAAR5 in humans is expressed not only in the olfactory system but also in certain brain structures, including the limbic regions receiving olfactory input and involved in critical brain functions. Thus, TAAR5 can potentially be involved in the pathogenesis of brain disorders and represents a valuable novel target for neuropsychopharmacology.

## 1. Introduction

Trace amine-associated receptors (TAARs) are G protein-coupled receptors (GPCRs) activated by the range of biogenic amines, with the majority of them (TAAR2–TAAR9) believed to be a class of olfactory receptors involved in sensing innate attractive and aversive odors [1,2,3]. In mice, 14 of the 15 TAARs (except TAAR1) are expressed in the main olfactory epithelium and project to the olfactory bulb glomeruli, which express the appropriate TAAR receptor. In humans, five out of six functional TAARs (TAAR2, TAAR5, TAAR6, TAAR8, and TAAR9) are believed to serve similar olfactory functions [4]. However, there is recent evidence from the knockout mouse model that at least one member of this family, TAAR5, is expressed also in the limbic areas of the brain receiving projection from the olfactory system and involved in the regulation of monoamine transmission, emotional behaviors, and adult neurogenesis [5,6]. In fact, TAAR5 was first identified in 1998 as a putative neurotransmitter receptor (PNR) and its mRNA was found at low levels in several human brain areas, including the amygdala, the hippocampus, the caudate nucleus, the thalamus, the hypothalamus, and the substantia nigra [7]. Appreciation of the olfactory function of TAAR2–TAAR9 took place significantly later [1] and the study of the human olfactory epithelium revealed that the TAAR5 receptor is the most highly expressed TAAR receptor in this tissue [8].

Human TAAR5 interacts with trimethylamine, which has an unpleasant fish odor, and, possibly, some other ligands produced by bacteria in spoiled food. Such ligands elicit aversive responses that prevent the ingestion of poisonous foods or foods harboring pathogenic microorganisms [9]. A population study revealed rare TAAR5 polymorphism that affects such aversion and leads to fish odor anosmia in carriers [10]. 3-iodothyronamine (T1AM), a thyronamine derivative that is known to activate TAAR1 [11], is now also considered an endogenous TAAR5 antagonist. It is assumed that T1AM is a feedback effector of thyroid signaling, effects of which, including hypothermia and cardiac depression, are opposite to those produced by thyroid hormone. Moreover, T1AM is known to be a modulator of monoaminergic transmission [12].

An evolutionary genetic survey demonstrated that TAAR5 is the most conserved TAAR subtype among all characterized mammalian species investigated so far [13]. Intriguingly, this receptor became the pseudogene in some primates [14] and its affinity for ligands varies in different species. For example, di- and trimethylamine display no agonistic activity for TAAR5 in most primates and cattle, but activate TAAR5 in mouse, rat, and dog [15]. Treatment with dimethylethylamine stimulates Gs signaling and downregulates the Gq/11 pathway for murine TAAR5, but not for human TAAR5. In contrast, T1AM application results in a robust reduction of MAPK signaling mediated by human TAAR5, but not by murine TAAR5 [16]. Generally, the human receptor is primarily activated by tertiary amines, but the murine TAAR5 ortholog is also activated by aromatic N-methyl piperidine and the secondary amine dimethylamine [17,18].

TAAR5 knock-out (TAAR5-KO) mice have revealed the involvement of this receptor in certain brain functions. The lack of TAAR5 in mice results in altered brain serotonin transmission and the anxiolytic or antidepressant-like phenotype [5], as well as elevated dopamine and its metabolite levels in the striatum, higher adult neurogenesis and dopaminergic neuron number [6]. Opposite effects were demonstrated in animals, treated with putative TAAR5 agonist α-NETA. This substance disrupts sensory information processing and alters brain electrophysiological activity in mice in a manner consistent with psychotic states [19,20,21,22]. Most importantly, prominent expression of TAAR5 was observed not only in the olfactory bulb but also in numerous limbic brain regions receiving olfactory input, such as the anterior olfactory nucleus, the olfactory tubercle, the orbitofrontal cortex, the amygdala, the hippocampus, the piriform cortex, the entorhinal cortex, the nucleus accumbens, and the thalamic and hypothalamic nuclei. Whether humans have a similar pattern of TAAR5 expression in the brain remains an open question.

Modern high-throughput methods provide a large amount of data stored in publicly available repositories. The aim of this study is the evaluation of TAAR5 expression in different areas in the human brain through mRNA expression datasets deposited online. Furthermore, we compared expression levels of TAAR5 in the brain in several neuropsychiatric disorders. No significant relationship between the TAAR5 gene SNP and schizophrenia, bipolar disorder, or fibromyalgia has been shown so far [12,23], but there is essentially no data currently on TAAR5 expression levels in neuropsychiatric disorders. These data are critical not only for understanding the role of TAAR5 in physiology and pathology but also for the development of future therapies based on targeting TAAR5.

## 2. Results

### 2.1. TAAR5 Expression in the Brain Cortical Areas and Hippocampus

The twenty-one of Affymetrix microarray-generated datasets listed in the Supplementary Table S1 and ten RNAseq-generated datasets listed in the Supplementary Table S2 represent TAAR5 expression in human neocortical structures. Hippocampus transcriptomic profiles are available in three microarray-generated datasets (Supplementary Table S1) and two RNAseq-generated datasets (Supplementary Table S2). TAAR5 cortical expression in all datasets is low and unstable. RNAseq-generated datasets predominantly demonstrate TAAR5 expression levels in cortex below 0.5 counts per million (CPM); only GSE68559 and GSE123496 datasets include specimens with TAAR5 expression above the cut-off. Meanwhile, TAAR5-positive samples consist only of nearly 30% of the study groups in these datasets. A similar expression pattern is demonstrated in the hippocampus, where TAAR5 is present in one of five cases in the GSE123496 dataset and 3 of 10 cases in the GSE68559 dataset. In the same datasets, DRD2, ADRB2, and HTR1A are expressed at low levels (i.e., 0.5–10 CPM) in the majority of neocortex and hippocampus samples.

Using log2 normalized expression >5.0 as the threshold, the most neocortical and hippocampal transcriptome microarray-generated datasets seem to include several TAAR5-negative specimens. In accordance with microarray data, TAAR5 expression is more often absent or detected at levels near threshold value that are lower than the expression of other well-studied GPCRs DRD2, HTR1A, or ADRB2 (Supplementary Figures S1 and S2).

**Figure 1:**
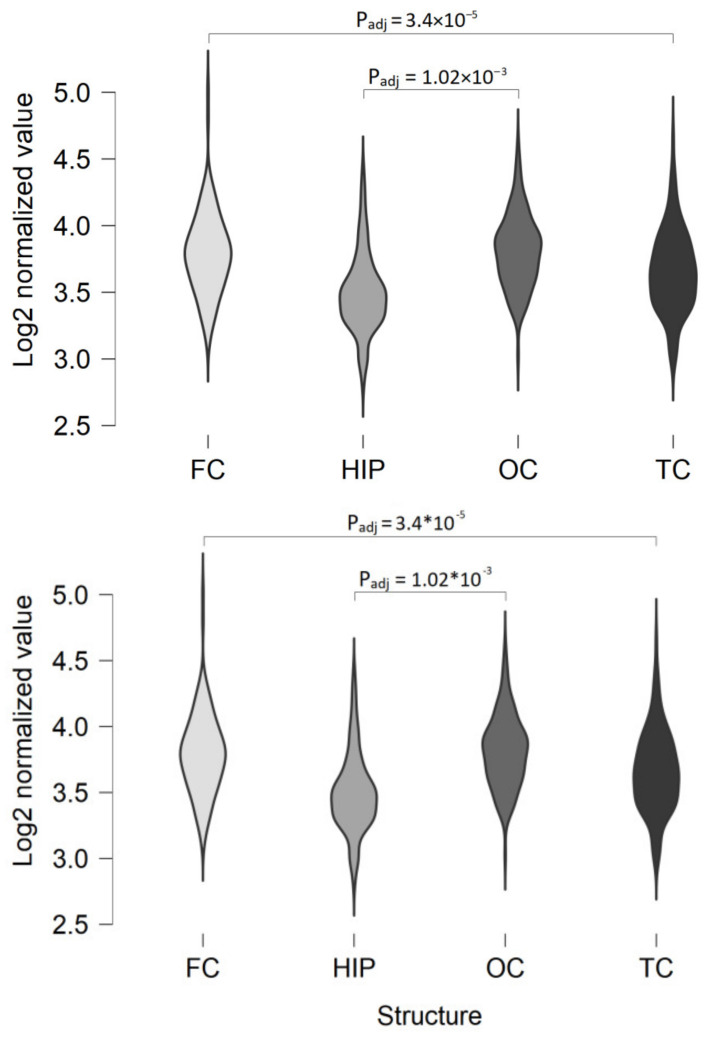
TAAR5 expression in different human cortex areas (GSE46706). FC—frontal cortex (*n* = 127), HIP—hippocampus (*n* = 122), OC—occipital cortex (*n* = 129), TC—temporal cortex (*n* = 119).

In the majority of datasets, TAAR5 expression levels are similar in the hippocampus and different neocortical areas, including the true isocortex (frontal cortex, parietal cortex, temporal cortex, occipital cortex), and periallocortex (including the insula and Brodmann area 24 area of the cingulate cortex). By comparison, in the GSE46706 dataset, generated by microarray on relatively large study groups including at least 119 specimens per cortical area, some differences between TAAR5 expression in the hippocampus and neocortex are identified. The hippocampal TAAR5 expression is lower than TAAR5 expression in other studied areas, but the difference reaches statistical significance only for the hippocampus and occipital cortex (P_adj_ = 1.03 × 10^−3^, Figure 1). The same study demonstrates the statistically significant difference of TAAR5 expression levels between the frontal cortex and temporal cortex (P_adj_ = 3.4 × 10^−5^).

TAAR5 expression in the cortical areas and hippocampus may be considered as low-positive. In the Human Protein Atlas (HPA) database [24], the highest TAAR5 protein expression in the brain was detected in the cortex and its consensus normalized expression (NX) level reaches 0.6 NX; that is, however, lower than the recommended threshold value 1.0 NX in this database. At the same time, no TAAR5 expression was detected in any neocortical area in the Allen Brain Atlas (ABA) [25] dataset (if the threshold value log2 = 5.0 is applied) or BrainSpan Atlas of the Developing Human Brain [26]. In the hippocampus, in the ABA dataset for the hippocampus, CA4 field samples were positive for TAAR5 mRNA in the microarray-based study but no TAAR5 expression was represented in the HPA.

**Figure 2:**
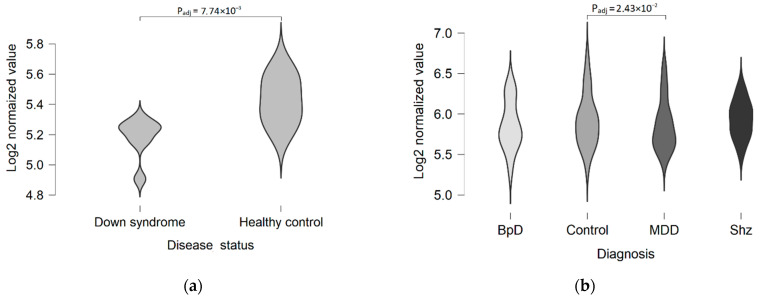
TAAR5 expression in the dorsolateral prefrontal cortex of healthy humans and patients with different neuropsychological or psychiatric conditions. (**a**) Downregulation of TAAR5 expression in Down syndrome patients (GSE5390, adult Down syndrome, *n* = 9, healthy controls, *n* = 6); (**b**) dysregulation of TAAR5 expression in patients with psychiatric disorders (GSE92538, bipolar disorder, *n* = 12, major depressive disorder, *n* = 29, schizophrenic, *n* = 31, control group, *n* = 55). BpD—bipolar disorder, MDD—major depressive disorder, Shz—schizophrenia.

TAAR5 expression in the prefrontal cortex may be deregulated in etiologically different pathologic conditions such as Down syndrome and major depressive disorder (Figure 2). No TAAR5 expression imbalance was found in the datasets which include cortical specimens from the patients with bipolar disorder (GSE5388, GSE5389, GSE53239, GSE80655), schizophrenia (GSE17612, GSE80655), Alzheimer’s disease (GSE53697), Huntington’s disease (GSE79666, GSE64810), Parkinson’s disease (GSE20295, GSE68719), multiple sclerosis (GSE123496), or frontotemporal lobar degeneration (GSE13162). Moreover, no differences were found between healthy controls and the patients with major depressive disorder in any other of the studied datasets that were generated to estimate gene expression disturbances in this disease (GSE54575, GSE54567, SE54568, GSE54570, GSE80655, and GSE101521).

### 2.2. TAAR5 Expression in the Amygdala and Basal Ganglia

Among the studies included in the analysis, eight microarray-generated datasets (Supplementary Table S1) and four RNAseq-generated datasets (Supplementary Table S2) represent the expression patterns in the basal ganglia. The general distribution of TAAR5, DRD2, ADRB2, and HTR1A minimal end maximal expression levels in the microarray-generated datasets are depicted in Supplementary Figure S2. The majority of microarray-generated datasets include at least one specimen with TAAR5 expression below the cut-off level. Three datasets include only TAAR5 negative tissues. In contrast, three other analyzed receptors are frequently expressed in all samples of the dataset and their maximal expression levels are two or more times higher than TAAR5 expression levels in linear scales.

The amygdala is the only TAAR5-expressing brain area in adult humans, according to BrainSpan Atlas of the Developing Human Brain. TAAR5 mRNA is also present in some amygdala samples included in HPA. In both datasets, TAAR5 expression is low, i.e., less than 0.001 RPKM in the Atlas of the Developing Human Brain and 0.4 NX or lower for the HPA dataset (such expression levels are designated as negative in the recommendations “explanation of the specificity category” for this database). Similarly, there are no loq2 normalized expression levels above the cut-off 5.0 detected in both microarray-generated datasets GSE54566 and GSE25219 that consist of amygdalar transcription profiles, excluding one case in the GSE25219 dataset. The more sensitive RNAseq method demonstrated TAAR5 expression at low levels (2.6 CPM or less) in 4 of 10 (40%) specimens in the GSE68559 dataset. All amygdala samples in this dataset were positive for DRD2, ADRB2, or HTR1A expression. The expression levels of these genes are low, i.e., 0.5–10 CPM, excluding two specimens with DRD2 medium expression levels 11–17 CPM.

The highest expression levels in basal ganglia in this study are identified in GSE35864 and GSE33010 microarray-generated datasets, which represent expression profiles in the caudate nucleus. In all specimens, the TAAR5 log2 normalized expression values were higher than the 5.0 cut-off level. At the same time, not one of the 58 caudate nucleus samples was TAAR5-positive in the GSE160521 RNAseq-generated dataset. All caudate nucleus samples in this dataset express DRD2 at the medium levels (33–116 CPM) and ADRB2 at low levels (1.5–5 CPM). HTR1A expression is below the cut-off in the majority of caudate nucleus samples in the set. TAAR5 also is accidentally detected in caudate nucleus samples included in the HPA dataset at the level 0.1 NX.

Putamen TAAR5 expression is negative in all specimens from the GSE46706 microarray-generated dataset and 31 of 35 (88%) in the GSE20295 microarray-generated dataset. In the RNAseq-generated dataset GSE160521, only one of 59 putamen samples is TAAR5 positive, with an expression level less than 0.1 CPM. In contrast, in the set of 10 putamen samples presented in the GSE68559 RNAseq-generated dataset, three samples are positive for TAAR5 expression with the levels up to 1.5 CPM. Therefore, TAAR5 expression in this region seems low and accidental. The DRD2, ADRB2, and HRT1A receptor expression patterns in the putamen specimens in the GSE160521 datasets are in general the same as in the caudate nucleus. In the GSE68559 dataset, DRD2 levels are low to middle, ADRB2 and HTR1A are expressed predominantly low.

**Figure 3:**
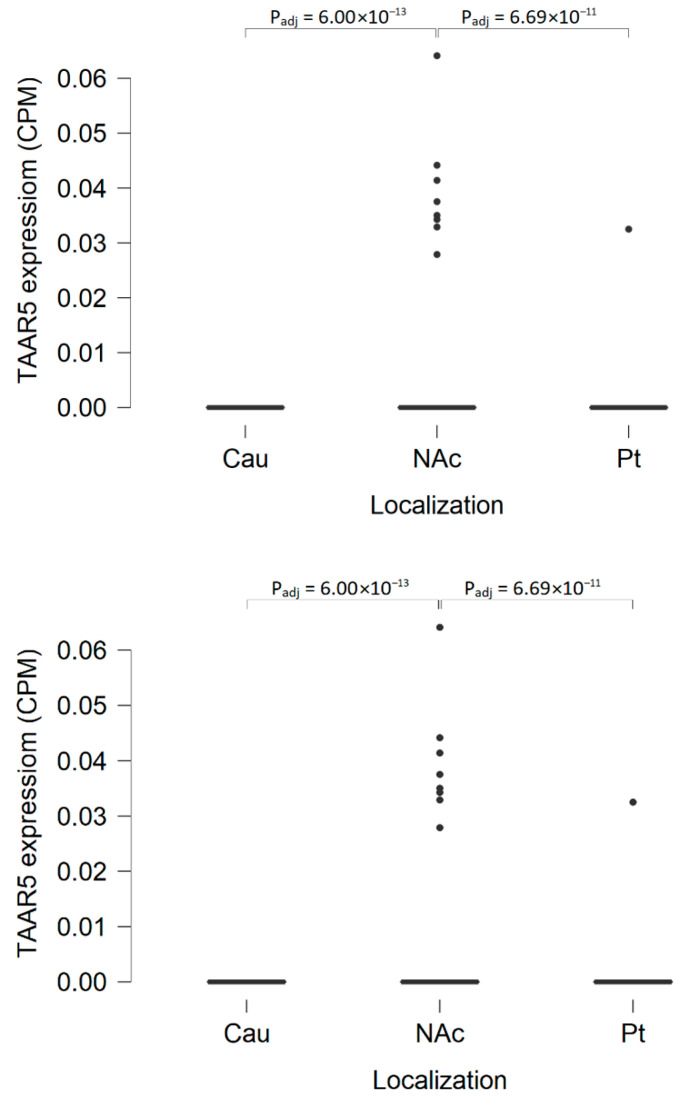
TAAR5 expression in the basal nuclei of healthy humans. TAAR5 expression in nucleus accumbens is significantly more pronounced compared to its expression in the caudate nucleus or putamen (GSE160521, caudate nucleus, *n* = 59; nucleus accumbens, *n* = 59; putamen, *n* = 59); Cau—caudate nucleus, NAc—nucleus accumbens, Pt—putamen.

TAAR5 expression in the nucleus accumbens is summarized in GSE160521 and GSE80655 RNAseq-generated datasets. GSE160521 demonstrates more pronounced TAAR5 expression in the nucleus accumbens than in the caudate nucleus (P_adj_ = 6.0 × 10^−13^) or putamen (P_adj_ = 6.69 × 10^−11^), but it was detected only in 9 of 59 (15%) specimens at the levels below 0.5 CPM (Figure 3). GSE80655 represents a generally similar TAAR5 expression pattern, 45% (10 of 22) of specimens in this dataset are TAAR5 positive, but the levels of TAAR5 expression are below 0.5 CPM. Both the GSE160521 and GSE80655 datasets represent medium DRD2 expression and low ADRB2 and HTR1A expression in nucleus accumbens (in the number of samples in the GSE160521 HTR1A expression is below the cut-off value of 0.5 CPM).

Some datasets include expression patterns in unspecified striatum regions. In the dorsal area of the striatum represented in GSE80336 TAAR5 expression below 0.5 CPM and sporadic (detected in 16% of samples, i.e., 3 of 18). The microarray-generated datasets GSE20295 and GSE54282 include expression profiles in non-specified parts of the striatum. No samples with log2 normalized expression levels above the cut-off 5.0 were present in these datasets.

No TAAR5 expression dysregulation in striatum is revealed in the patients with bipolar disorder (GSE80336, GSE80655), major depressive disorder (GSE80655), schizophrenia (GSE80655), or HIV-associated neurological disorders (GSE35864). Major depressive disorder also did not impact the TAAR5 expression in the amygdala (GSE54564).

### 2.3. TAAR5 Expression in the Diencephalon Structures

In accordance with ABA recommendations [25], TAAR5 expression is discretely detected in some parts of the diencephalon, including the arcuate nucleus and paraventricular nucleus of the hypothalamus, anterior lateral hypothalamic areas, the dorsal lateral geniculate nucleus of the thalamus, and zona incerta. In contrast, no TAAR5 expression is detected in hypothalamic specimens in the HPA dataset.

Three microarray-generated Gene Expression Omnibus (GEO) datasets that present the diencephalon expression profiles are available, including data generated by microarray study of the human hypothalamus (GSE1147) or thalamus (GSE46706, GSE25219). Using the threshold log2 value = 5.0, at least 47% of samples were positive for TAAR5 expression. It was not specified what part of the hypothalamus was examined [27], so it is impossible to compare this result with ABA data. All hypothalamic specimens were positive for DRD2 and ADRB2 expression, but only in 39% of them, HTR1A expression was above the cut-off log2 normalized value = 5.0. No specimens with TAAR5 expression log2 normalized levels above 5.0 were found in both datasets for thalamus, possibly because of the sampling outside the areas with pronounced TAAR5 expression. DRD2 was expressed in all thalamic specimens, and more than half of specimens were positive for ADRB2 expression, but HTR1A expression values were controversial, with only one positive specimen in the GSE46706 (<1%) and 30% positive cases in GSE25219 using the cut-off log2 normalized value = 5.0.

### 2.4. TAAR5 Expression in the Brain Stem and Cerebellum

Data from ABA demonstrate the heterogeneity of TAAR5 expression in the cerebellum, with the highest expression in the nucleus globosus and fastigial nucleus. The TAAR5 expression in the cerebellum samples included in the HPA dataset was very weak (i.e., 0.1 NX, that is 10 times lower than recommended cut-off value for this database).

In the GEO repository, cerebellum expression is represented by microarray-generated datasets (GSE13162, GSE167447, GSE35974, GSE44971, GSE25219, GSE46706) and a single RNAseq-generated dataset (GSE68559). Both microarray datasets and RNAseq-generated GSE68559 demonstrate low TAAR5 expression. Only 3 of 10 cerebellar specimens are positive for TAAR5 expression, and it reaches levels of 0.7–2.3 CPM in GSE68559. The expression of DRD2, ADRB2, and HTR1A also varies from the values near zero to the levels over 5 CPM for HTR1A, and over 100 CPM for DRD2 and ADRB2. In microarray datasets, TAAR5 log2 normalized expression values are frequently below 5.0 both for minimal and maximal expression levels in the datasets. For DRD2, ADRB2, and HTR1A, minimal expression log2 normalized values were also below 5.0, but the maximal expression values of these GPCRs seem more frequently above this cut-off than TAAR5 maximal expression values (Supplementary Figure S3). No significant influence of frontotemporal lobar degeneration on TAAR5 expression in the cerebellum was defined in the GSE13162 dataset.

Seven microarray-generated expression datasets (Supplementary Table S1) and one RNAseq-generated dataset GSE136666 represent TAAR5 expression in the substantia nigra. The TAAR5 expression levels reach only 0.04 CPM and present at the level higher than zero only in 40% (two of five) of the samples in RNAseq-generated data. Expression of DRD2 in the same samples may be interpreted as lower to middle (i.e., 7.6–17.5 CPM), other receptors were expressed at low levels (ADRB2 2.8–3.9 CPM, HTR1A 0.08–3.3 CPM). A similar expression distribution is reflected in microarray-generated datasets. The majority of these series include specimens with low expression of all receptors (i.e., Log2 normalized expression values below 3). The maximal expression values for DRD2, ADRB2, and HTR1A are four times over TAAR5 expression values in linear scales or higher (Supplementary Figure S4). Weak positive TAAR5 expression in the substantia nigra also was demonstrated in the ABA.

There is limited evidence of TAAR5 expression in the other parts of the brain stem. RNAseq data from the GSE68559 dataset demonstrate TAAR5 expression in 45% (four of nine) raphe nuclei specimens, in which the expression levels were 0.5–4.1 CPM. Expression of DRD2 and ADRB2 in the same dataset was interpreted as low or middle (i.e., 0.6–15.0 CPM for DRD2 excluding one specimen with undetectable expression and two specimens with expression levels below the cut-off value of 0.5 CPM, and 0.52–16.2 CPM for ADRB2, excluding one specimen with the expression levels below the cut-off value of 0.5 CPM). HTR1A receptor expression may be interpreted as low, i.e., 0.5–8.3 CPM, excluding one HTR1A-negative specimen. The ABA dataset includes TAAR5 positive samples of some midbrain nuclei, including the Edinger–Westphal nucleus, oculomotor nucleus complex, interstitial nucleus of Cajal, and cuneiform nucleus in the reticular formation. At the same time, following the HPA data, TAAR5 expression presents in a few midbrain specimens at a low level that reaches only 0.1 CPM.

### 2.5. TAAR5 Expression in the White Matter

Only four GEO datasets, including RNAseq-generated GSE138614, GSE123496, and microarray-generated GSE35864, GSE46706, are available in the GEO repository. In GSE138614 TAAR5 expression is present in 32% (8 of 25) of white matter samples from healthy subjects. In all cases, the TAAR5 expression levels are 0.2 CPM or lower. GPCRs DRD2, ADRB2, and HTR1a are expressed at low levels (0.5–10 CPM) in the majority of samples. In the internal capsule, some specimens demonstrate significantly higher DRD2 expression levels, reaching 76 CPM (medium expression level). TAAR5 is expressed in corpus callosum specimens in the GSE123496 dataset, in which all five specimens were positive for TAAR5 expression with CPM values of 0.3–0.7. In the GSE46706 dataset, log2 normalized fluorescence values were below the cut-off 5.0, but in GSE35864, TAAR5 expression was more pronounced and all log2 normalized values for it were >6.4.

**Figure 4:**
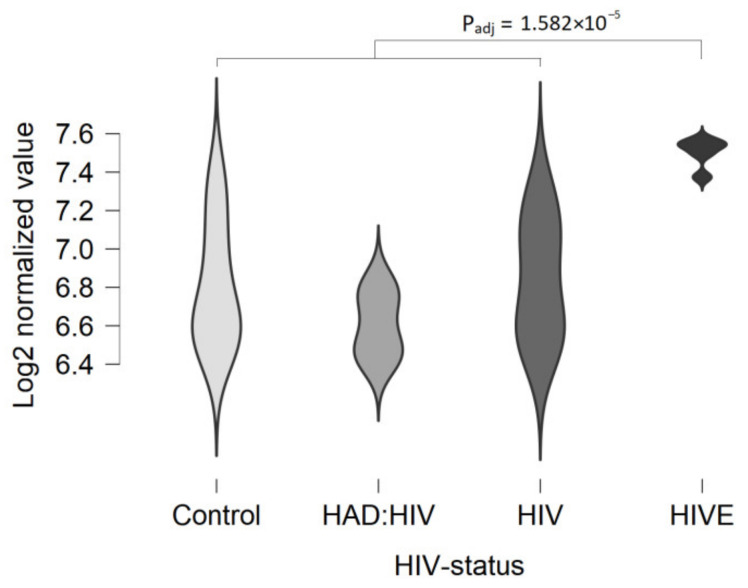
TAAR5 expression up-regulation in the white matter in patients with HIV-associated encephalitis (microarray-generated dataset GSE35864). Control—non-HIV-infected subjects (*n* = 6), HAD:HIV—HIV-infected subjects with HIV-associated dementia (*n* = 7), HIV—HIV-infected subjects (*n* = 6), HIVE— subjects with HIV encephalitis (*n* = 6).

There is significant up-regulation of TAAR5 in white matter in humans with HIV-associated encephalitis (HIVE), as demonstrated by the microarray hybridization in the GSE35864 dataset. No differences were found in the healthy controls, HIV-infected patients without neurological signs, and patients with HIV-associated dementia (Figure 4). This data supports the assumption of TAAR5 expression in white matter, at least in some pathologic conditions. In contrast, in the GSE138614 and GSE123496 datasets, a similar frequency of TAAR5 expression is detected in healthy controls and patients with multiple sclerosis. The expression levels are slightly higher in multiple sclerosis patients and reach 0.7 CPM, but the up-regulation is not statistically significant.

In general, TAAR5 expression in the human brain may be considered as low-positive, so the possibility of its detection and estimation may depend on technical parameters such as the experimental design or mRNA concentration in the sample.

### 2.6. TAAR5 Expression at the Cell Level and Possible Sources of Bias

**Figure 5:**
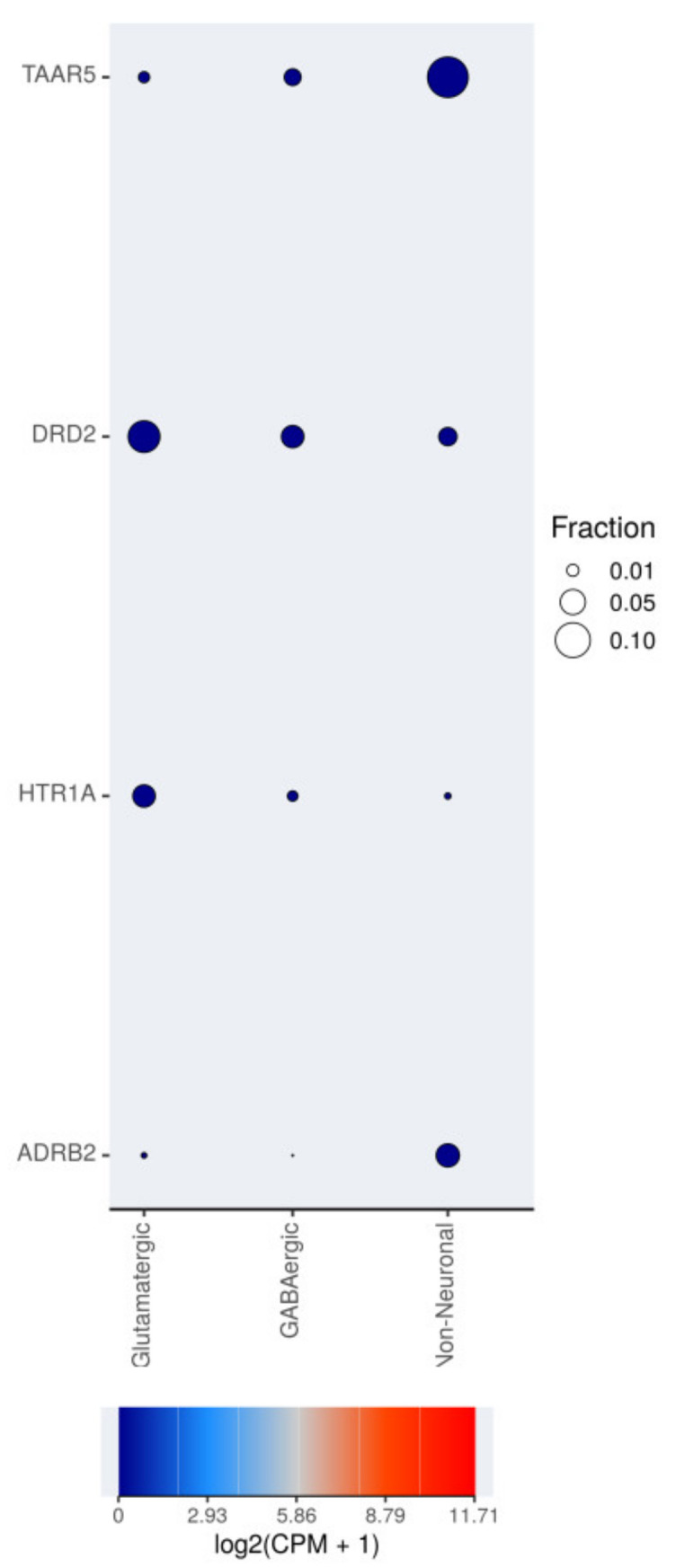
Single-nuclei TAAR5, DRD2, HTR1A, and ADRB2 expression in different cell types in human middle temporal gyrus. RNA-sequencing data are available on the RNAseq Data Navigator (http://celltypes.brain-map.org/rnaseq/human (accessed on 10 March 2021, [28]); the figure was generated by the RNAseq Data Navigator interactive web interface. Fraction is the fraction of cells that are positive for mRNA expression.

The expression of TAAR5, DRD2, HTR1A, and ADRB2 mRNAs in different cell types of the medial temporal gyrus was estimated in single-nuclei RNAseq available on the Allen Cell Types Database. All four targets are expressed at extremely low levels in all cell types, i.e., glutamatergic and hypothalamic gamma-aminobutyric acid (GABA)-ergicneurons, and different types of glial cells. The detected expression levels converted to log2 (CPM+1) are close to zero in almost all positive cases. At the same time, the distribution of TAAR5 and other GPCR expression in different cell populations is not homogenous. TAAR5-positive cells are predominant in the non-neuronal population (about 10%). Only 1% of glutamatergic neurons and 5% of GABAergic neurons express TAAR5 in the examined dataset. ADRB2 receptor expression also seems more frequently detected in non-neuronal cells, in contrast, DRD2 and HTR1A expression predominate in neuronal populations, especially in glutamatergic neurons (Figure 5). So, in the brain specimens, TAAR5 expression levels may be significantly biased by non-neuronal cells.

**Figure 6:**
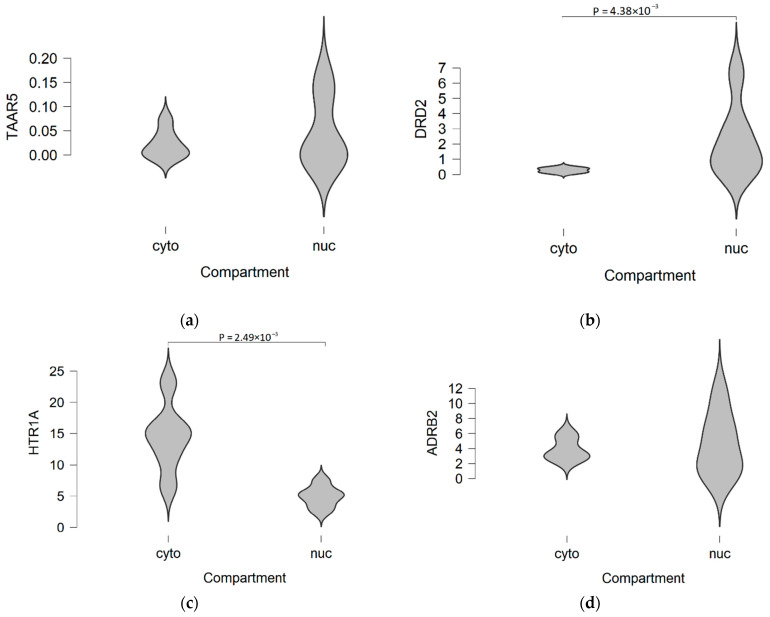
TAAR5, DRD2, HTR1A, and ADRB2 expression in cytoplasmic and nuclear fractions of healthy adult frontal cortex specimens in the RNAseq-generated dataset GSE110727. (**a**) TAAR5 mRNA distribution in cytoplasmic and nuclear compartments; (**b**) DRD2 mRNA distribution in cytoplasmic and nuclear compartments; (**c**) HTR1A mRNA distribution in cytoplasmic and nuclear compartments; (**d**) ADRB2 mRNA distribution in cytoplasmic and nuclear compartments. Cito–cytoplasmic expression, nuc—nuclear expression, data represented in CPM values.

As it is known, mRNAs are not concentrated in the nuclear compartment of neurons but distributed in the cytoplasm both in the soma and the remote cell parts, such as axonal synapses. Thus, the differences of TAAR5, DRD2, HTR1A, and ADRB2 mRNA quantities between cytoplasmic and nuclear fractions of homogenized brain tissue were estimated in the RNAseq-generated dataset GSE110727. The expression levels of TAAR5 are extremely low in this dataset. The TAAR5 transcripts present both in nuclear and cytoplasmic fractions (Figure 6a). DRD2 expression was significantly higher in the nuclear fraction, in contrast, HTR1A expression was more pronounced in the cytosolic fraction. For DRD2 and HTR1A, expression differences reach statistical significance (P_adj_ < 0.01, Figure 6b,c). ADRB2 mRNA was represented in nuclear and cytoplasmic fractions at the same levels. Nuclear ADRB2 levels were a little more variable than cytoplasmic levels (Figure 6d). In the original study for which this dataset was generated, similar results were demonstrated by the different statistical processing (i.e., DESeq2’s normalization in the original study vs EdgeR’s CPM trimmed mean of M values in the present study) [29]. Such distribution of GPCR mRNAs in neurons makes difficult interpretation of single-nuclei RNAseq data for these targets.

## 3. Discussion

While the expression of TAAR5 in the olfactory sensory neurons and the olfactory bulb is well established [4,5], the expression of TAAR5 in the brain was also described previously by different approaches. When the TAAR5 receptor was first reported as PNR, its expression was evidenced by Northern blot hybridization in the different areas of the human brain, including the amygdala, hippocampus, caudate nucleus, thalamus, and hypothalamus. A weak expression signal was also found in the substantia nigra [18]. In situ hybridization signals specific to TAAR5 RNA were detected in the mouse arcuate nucleus, the ventromedial hypothalamus, and the amygdala [16]. Analysis of LacZ histochemical staining on brain sections in TAAR5-KO mice with LacZ reporter gene inserted in the place of the TAAR5 gene demonstrated LacZ labeling in the olfactory bulb, the amygdala, the orbitofrontal cortex, the CA1 area of the hippocampus, the anterior olfactory nucleus, the piriform cortex, the thalamic region, the nucleus accumbens, the entorhinal cortex and the ventromedial nucleus of the hypothalamus [5]. According to recent transcriptomic analysis, all TAARs are expressed at low levels in the cortical and limbic areas, the basal ganglia, the cerebellum, and the brainstem of human brains, with the most highly expressed receptor being TAAR5; however, no data on patterns of individual TAARs expression were presented in this report [30].

High-throughput transcriptomic studies described here also provided evidence for the wide distribution of TAAR5 expression in the adult human brain, particularly in the limbic brain areas involved in olfactory information processing and responsible for emotional regulation. The unique localization of TAAR5 in the olfactory epithelium and the olfactory bulb [1,3,4,31] as well in the downstream “emotional” limbic areas receiving input from the olfactory bulb, such as the frontal cortex, the amygdala, the hippocampus, the nucleus accumbens, the thalamus, the hypothalamus, suggest the intriguing possibility that “olfactory” TAAR5-mediated brain circuitry may represent a new type of neurotransmitter system involved in the transmission of innate odors into emotional behavioral responses [5] and adult neurogenesis [6]. Expression of TAAR5 mRNA was also detected in various structures of the basal ganglia, the cerebellum, the substantia nigra, and the white matter. It would be important to clarify the functional role of TAAR5 in these brain areas in future studies. Intriguingly, an elevated number of tyrosine hydroxylase-positive dopaminergic neurons in the substantia nigra and an increased adult neurogenesis were found in mice lacking TAAR5 [6]. It would be important also to explore if other human “olfactory” TAARs will be acting as TAAR5 by being expressed in limbic brain areas involved in the regulation of emotional behaviors. In fact, studies in TAAR2-KO mice revealed a similar, but not identical, pattern of brain TAAR2 expression in limbic areas, as well as alterations in classical monoamines levels and emotional behaviors [32].

At the same time, in the majority datasets, TAAR5 expression is low and sporadically detected only in the part of the studied populations. Such expression pattern was detected in different areas of the cortex, the hippocampus, and also in the amygdala, the caudate nucleus, the nucleus accumbens, the thalamus, the hypothalamus, the cerebellum, and the substantia nigra. The mined data that support this conclusion is briefly described in Table 1.

Transcriptomic datasets reviewed in this paper were obtained in different laboratories and cannot be standardized, thus somewhat impeding their interpretation. The certain measures to filter and normalize the data discussed in the section “Material and Methods” do not provide full uniformity of the data and the batch effects overcoming. These imperfections of public data may be a source of biases in this study. In addition, multiplex high-throughput methods are characterized by imperfections that can make some result controversial or difficult to interpret. These biases also are listed in Table 1. The most significant of them is the difficulty of data interpretation in microarray-generated datasets and unstable sample quality in the RNAseq-generated datasets.

Well-studied monoaminergic receptors, including DRD2, ADRB2, and HTR1A, are expressed in most brain areas analyzed. Cortical expression and binding activity of DRD2 were demonstrated in human [33,34], and different model animals, including rat [35], mice [36], rhesus monkeys [37]. The cortical activity of the ADRB2 receptor was demonstrated in human [38]. 5-HT1A receptor mRNA also was found in the hippocampus [39] and other cortex areas [40]. Following this data, our meta-analysis demonstrates stable expression of the listed receptors in different areas of the cortex. Generally, TAAR5 expression is weaker and less stable than the expression of DRD2, ADRB2, and HTR1A in the same areas. Similar expression patterns were detected in other brain structures, which demonstrate the extremely low levels of TAAR5 in the brain and low or medium expression of other receptors. Nevertheless, in some regions and datasets, differences between the expression of these GPCRs with well-established brain localization and TAAR5 were not so dramatic and comparable, with few cases demonstrating even lower expression than TAAR5. Due to trace levels of TAAR5 mRNA in the many tissues, it seems plausible to apply the commonly used cut-offs with precaution and take into account the expression values below these cut-offs.

Cell composition of the tissues is complex and single-cell RNA sequencing is an attractive way to better understand the biological processes. For the nervous tissue, single-nuclei sequencing is commonly used instead of single-cell sequencing. The single-nuclei RNAseq data for the brain cortex demonstrate that, in the cortical structures, TAAR5 expression predominates in the non-neuronal cells. On the other hand, the nuclear fraction of the transcriptome data does not entirely represent the expression pattern in the whole neuron. mRNA presented in the neuronal cytoplasm, both in soma and neurites. Poly(A) RNA colocalized with ribosomes and elongation factor 1a (EF1a) are non-stochastically distributed into neurons to provide protein syntheses at the appropriate sites, including dendrites and axons [41,42]. In our meta-analysis, we include the dataset GSE110727 that demonstrates TAAR5 transcripts equally distribute in nuclear and cytoplasmic compartments in the frontal cortex. The original study for which this dataset was generated demonstrated 5109 transcripts that were significantly more abundant in the cytosol and 5397 transcripts significantly more abundant in the nucleus [27]. In some other brain structures, TAAR5 may be also heterogeneously distributed in the cells, as it was demonstrated for monoaminergic receptors DRD2 or ADRB2 in the present dataset.

Results of animal studies indicate that TAAR5 might be involved in the pathogenesis of neuropsychiatric diseases. TAAR5-KO mice demonstrate anxiolytic/antidepressant-like phenotype [5]; at the same time animals treated with non-selective TAAR5 agonist α-NETA have disrupted synchronization of cortical gamma rhythms [22] and deregulated sensory gating [20,43] mirroring pathological changes in schizophrenia. Several studies included in the meta-analysis were devoted to demonstrating the transcriptome dis-balances in schizophrenia, bipolar disorder or major depressive disorder, but only one demonstrated significant downregulation of TAAR5 in the prefrontal cortex in major depressive disorder. No TAAR5 expression disbalance was demonstrated in bipolar disorder or schizophrenia patients in the same dataset. Major depressive disorder may lead to reductions in the volume of the prefrontal cortex, suggesting atrophy and disruption of connectivity [44]. TAAR5 downregulation in the prefrontal cortex area also was demonstrated in Down syndrome. The loss of cortical projection neurons in locus coeruleus (norepinephrinergic), dorsal raphe nuclei (serotonergic), and ventral tegmental area (dopaminergic) is the possible cause of monoaminergic systems impairments in this disease [45,46,47]. It would be interesting to explore if TAAR5-mediated dysregulation of monoaminergic transmission can contribute to the pathogenesis of these conditions.

Progression of HIV-associated brain impairment to HIVE is accompanied by the growth of HIV RNA load that is three log2 units higher in the HIVE group [48]. Genes associated with immune response, defense response, cytokine and interferon response, and antigen presentation are up-regulated in the white matter of HIVE patients [49]. Infiltration with B-cells distinguishes HIVE brain from healthy human or HIV-infected without HIVE patient brain. The possibility that upregulation of white matter TAAR5 in HIVE patients may be due to encephalitis-related infiltration of B-cells that are known to express TAAR5 [50] should be investigated.

The provided data shed light on the distribution of TAAR5 expression in the human brain. These findings are not sufficient to fully appreciate TAAR5’s functional significance as well as its potential involvement in the pathogenesis of neuropsychological diseases. At the same time, the evidence of the presence of TAAR5 in the human brain structures enables the translation of accumulating knowledge about the functional role of this receptor [6,19,20,21,22,43] from model animals to humans.

In conclusion, TAAR5 is ubiquitously expressed in many human brain structures with predominant localization in limbic brain areas involved in olfactory information processing and emotional regulation. In most brain regions, its expression is low and seems unstable, perhaps because of its limited distribution in select cell types. Inappropriate specimen quality or study design in some publicly available datasets also might be a confounding fact. At the same time, TAAR5 expression may be deregulated in some pathological conditions due to its possible implication into pathogenesis or compensatory reactions. More in-depth investigations of TAAR5’s involvement in human physiology and pathology may open up new avenues for the development of novel therapeutic approaches for neuropsychiatric disorders.

## 4. Materials and Methods

### 4.1. Data Collection and Inclusion Criteria for Datasets

Publicly available transcriptome datasets were retrieved from GEO repository [51]. First, the GEO browser available on http://www.ncbi.nlm.nih.gov/geo/browse/ (accessed on 5 July 2021) [52] was searched for the terms: “brain”, “striatum”, “dentate gyrus”, “thalamus”, “hypothalamus”, “white matter”, or “choroid plexus”. Datasets were included into review if suitable according inclusion criteria:

Inclusion criteria for microarray-generated datasets

- Taking into account low TAARs expression, datasets with small sample size were excluded to prevent sampling bias. The inclusion criteria were at least 5 samples from healthy adult subjects per structure in the dataset.
- To escape the incorrect data comparison, the exact structure definition was applied as the inclusion criteria. Datasets with samples descriptions such as “brain sample” (with no any explanations of the studied brain part) or “cortex” (without clarifications such as “frontal cortex” or “insula”, etc.) were excluded.
- Availability of log2 or non-logged expression values, including TAAR5 expression values.
- As the majority of microarray-generated datasets were acquired on Affymetrix platforms, we excluded datasets generated on other kinds of microarray to include only comparable data in the review.

Inclusion criteria for RNAseq-generated datasets

- At least 5 samples from healthy adult subjects per structure in the dataset.
- The exact structure definition (same as for microarray-generated datasets).
- Because of the low TAARs mRNA transcription, its expression patterns may be estimated by RNAseq only if appropriate sequencing depth is applied. To prevent overload with false-negative results, at least 20 million reads in SRA file for each run, or mean reads number in SRA files in the dataset 40 million or higher, were applied as the threshold for dataset selection.
- Availability of any quantitative data for TAAR5 expression in Supplementary Materials (count table or other attached data tables).

After exclusion of non-relevant datasets, 31 microarray-generated datasets and 14 NGS-generated datasets were selected for future analysis (Supplementary Tables S1 and S2, respectively).

### 4.2. Microarray-Generated Datasets Analysis

Gene expression in microarray-generated transcriptome datasets was estimated by the Phantasus available at http://www.artyomovlab.wustl.edu/phantasus/ (accessed on 20 March 2021) [53] Prior to the analysis, the datasets were log2 transformed if necessary.

Differential TAAR5 expression analysis was performed to compare its expression in different brain structures (for example in different cortical areas or different basal ganglia) or in specimens from neurologically/mentally healthy individuals and patients with different neurological or psychiatric conditions (in accordance with the design and annotation of datasets). Differentially expressed genes were identified by the limma R package [54]. Raw *p* values were adjusted for multiple testing using the Benjamini–Hochberg procedure and only adjusted P (Padj) values higher than 0.05 were considered significant.

Since datasets based on platforms GPL570 ([HG-U133_Plus_2] Affymetrix Human Genome U133 Plus 2.0 Array) and GPL5175 ([HuEx-1_0-st] Affymetrix Human Exon 1.0 ST Array [transcript (gene) version]) are not available in the Phantasus, they were analyzed using GOE2R interactive web-based interface available at http://www.ncbi.nlm.nih.gov/geo/geo2r/ (accessed on 20 March 2021 [55]). A forced normalization option was used to normalize microarray data before analysis and differential expression was estimated. *p* values were adjusted using the Benjamini–Hochberg procedure.

To elucidate the level of TAAR5 expression in microarray-generated datasets, we compared its expression with three other GPCRs well known to be expressed in the brain: dopamine D2 receptor (DRD2), serotonin 5-HT1A receptor (HTR1A), and beta-2 adrenergic receptor (ADRB2). Minimal and maximal expression values for TAAR5, DRD2, HTR1A, and ADRB2 were extracted from the “profile graph” tab.

We used 5.0 as the cut-off value for log2 normalized gene expression according to previous studies [56,57,58].

Plots were created in free desktop JASP statistical software (JASP, Amsterdam, Netherlands) [59].

### 4.3. RNAseq-Generated Datasets Analysis

All RNA sequencing datasets excluding GSE110757, GSE138614, GSE160521, and GSE80655 were analyzed by online GREIN console (available at http://www.ilincs.org/apps/grein/?gse= (accessed on 23 March 2021) [60,61]. Prior to the statistical analysis, all datasets were transformed to CPM values. Minimal and maximal TAAR5 expression values were extracted from the counts table tab panel. Differential expression was estimated by the “create signature” function. *p*-values were adjusted for multiple testing correction using the Benjamini–Hochberg method.

Datasets GSE110757, GSE138614, GSE160521, and GSE80655 are not available on the GREIN and were analyzed in a different way. Count matrices for these datasets were downloaded from its supplementary data on GEO Accession Display. Data were CPM transformed and trimmed means of Mnormalized by the Bioconductor R package edgeR, then differential expression was estimated by the same package [62]. *p*-values were adjusted for multiple testing correction using the Benjamini–Hochberg method. Only Padj values higher than 0.05 were considered significant.

To interpret numerical values, we used Expression Atlas terms [63], i.e., expression level is below cut-off (<0.5 CPM), low expression level if CPM between 0.5 to 10, medium expression level if CPM between 10 and 1000. Over 1000 CPM may be interpreted as high expression level, but so pronounced expression is not typical for GPCRs and was not identified in this study.

Plots were created in free desktop JASP statistical software (JASP, Amsterdam, Netherlands) [59].

### 4.4. Allen Brain Atlas Datasets

Allen Brain Atlas (ABA, available at http://www.human.brain-map.org/ (accessed on 23 December 2020)) [25] and BrainSpan Atlas of the Developing Human Brain (available at http://www.brainspan.org/ (accessed on 23 December 2020)) [26] data for TAAR5 expression were obtained by Bioconductor R packages ABAdata and ABAEnrichment [64]. As ABA data were obtained by Agilent 4x44 Whole Human Genome array, a log2 expression threshold = 5 was chosen to distinguish TAAR5-positive and TAAR5-negative brain areas.

The medial temporal gyrus single-nuclei sequencing dataset was analyzed and the dot-plot diagram was generated in the RNAseq Data Navigator (http://celltypes.brain-map.org/rnaseq/human (accessed on 10 March 2020) interactive web-based interface of the Allen Cell Types Database (2015) [28]. Expression levels were included in the review in log2(CPM+1), as presented in the dataset.

### 4.5. Human Protein Atlas

Human Protein Atlas (HPA) data were received directly from a web resource available at http://www.proteinatlas.org (accessed on 28 December 2020) [24]. Expression levels were included in the review in consensus normalized expression value (NX), as provided in the database.

## Figures and Tables

**Table 1:** Evidence for TAAR5 expression in brain mined by high-throughput data analysis.

Structure	Microarray	RNAseq	Public Databases
Cortical areas: frontal cortex, parietal cortex, temporal cortex, occipital cortex, cingulate cortex, and insula.	The expression level is over the cut-off value in some specimens (log2 values > 5.0). Moreover, some statistically significant variations between cortex areas or health conditions are detected.	In several datasets, some samples are true positive for TAAR5 expression (CPM > 0.5).	HPA data demonstrated accidental TAAR5 expression below the cut-off recommended for this database.
Hippocampus	The expression level is over the cut-off value in some specimens (log2 values > 5.0).	30% (3 of 10) true positives for TAAR5 expression (CPM > 0.5) in GSE68559.	In the ABA, CA4 field samples may be considered positive.
Amygdala		In several datasets, some samples are true positives for TAAR5 expression (CPM > 0.5)	Positive TAAR5 expression is demonstrated in the BrainSpan Atlas of the Developing Human Brain and HPA (below cut-off).
Nucleus accumbens	More pronounced TAAR5 expression in the nucleus accumbens than in the caudate nucleus in GSE160521 (taking into account that TAAR5 is expressed at the levels below 0.5 CPM).		
Thalamus			In the ABA, some thalamic structures may be considered positive.
Hypothalamus	The expression level is over the cut-off value in some specimens (log2 values > 5.0).		In the ABA, some hypothalamic structures may be considered positive.
Striatum	The expression level is over the cut-off value in some specimens (log2 values > 5.0).	In several datasets, some samples are true positive for TAAR5 expression (CPM > 0.5).	
Cerebellum	The expression level is over the cut-off value in some specimens (log2 values > 5.0).	In several datasets, some samples are true positive for TAAR5 expression (CPM > 0.5).	HPA data demonstrated accidental TAAR5 expression below the cut-off recommended for this database. In the ABA, some cerebellar areas may be considered positive.
Substantia nigra	The expression level is over the cut-off value in some specimens (log2 values > 5.0).		Positive expression was detected in the ABA dataset.
White matter	The expression level is over the cut-off value in some specimens (log2 values > 5.0). The up-regulation of expression in HIV-associated encephalitis is statistically significant.	In several datasets, some samples are true positive for TAAR5 expression (CPM > 0.5).	
Source of bias	The method is not adopted to confirm the absence or presence of mRNA, is not the purpose is the differential gene expression examination.	The sensitivity of the study depends on its design. In some datasets, the number of reads in the SRA file varies from 20 to 100 million. Such datasets are highly heterogeneous. Samples that include only 20 million reads may be inadequate to estimate low-expressed genes.	Data were received by RNAseq or microarray and are not free from corresponding weaknesses.

## Data Availability

All of the data is presented in the article and Supplementary Materials. No additional data is reported.

## Supplementary Materials

The following are available online at https://www.mdpi.com/article/10.3390/ijms22168802/s1.
