# Supplementary material for: Pattern of TAAR5 Expression in the Human Brain Based on Transcriptome Datasets Analysis
Source: Int J Mol Sci. 2021 Aug 16;22(16):8802. doi: 10.3390/ijms22168802 (PMC8395715; doi:10.3390/ijms22168802)
Supplement: Supplementary file 1 [file ijms-22-08802-s001.zip › ijms-1323173-supplementary.pdf]

Article

Pattern of TAAR5 expression in the human brain based on transcriptome datasets analysis

Anastasia N. Vaganova[1], Ramilya Z. Murtazina[1], Taisia S. Shemyakova[1], Andrey D. Prjibelski[1], Natalia V. Katolikova[1] and Raul R. Gainetdinov[1,2,*]

[1]Institute of Translational Biomedicine, St. Petersburg State University, Universitetskaya nab. 7/9, 199034, St. Petersburg, Russia.
[2]St. Petersburg University Hospital, St. Petersburg State University, St. Petersburg, Russia, Institute of Translational Biomedicine, St. Petersburg State University, Universitetskaya nab. 7/9, 199034, St. Petersburg, Russia.
*Author to whom correspondence should be addressed

SUPPLEMENTARY MATHERIALS

Supplementary Table S1. Brain RNA expression datasets (microarray data)

GSE identifier	Authors	Title	Structure*	Platform	Analytic software
GSE1147	Rinn JL et al, 2004	Sex-Specific Transcription in Human Hypothalamus	Hypothalamus	Affymetrix Human Genome U133A Array	Phantasus
GSE13162	Chen-Plotkin AS et al, 2008	Expression data from postmortem human brain samples with and without FTLD-U	Cerebellum, frontal cortex	Affymetrix Human Genome U133 Plus 2.0 Array	Phantasus
GSE167447	Taylor MD, 2021	Expression data from normal human cerebellum	Cerebellum	Affymetrix Human Gene 1.1 ST Array	Phantasus
GSE17612	Maycox PR et al, 2009	Comparison of post-mortem tissue from brain BA10 region between schizophrenic and control patients.	Prefrontal cortex	Affymetrix Human Genome U133 Plus 2.0 Array	Phantasus
GSE20295	Middleton FA, 2010	Transcriptional analysis of multiple brain regions in Parkinson's disease	Substantia nigra, putamen, prefrontal cortex	Affymetrix Human Genome U133 Plus 2.0 Array	Phantasus
GSE20333	Edna G et al, 2010	Gene expression profiling of parkinsonian substantia nigra	Substantia nigra	Affymetrix Human HG-Focus Target Array	Phantasus
GSE25219	Kang H et al, 2010	Spatio-temporal transcriptome of the human brain	Cerebellar cortex, thalamus, amygdala, striatum, hippocampus, different cortical areas	Affymetrix Human Exon 1.0 ST Array	GEO2R
GSE33010	Zeng J et al, 2011	Human-specific patterns of gene expression in the brain (Arrays)	Caudate nucleus, hippocampus	Affymetrix Human Genome U133 Plus 2.0 Array	Phantasus
GSE35864	Gelman BB, 2012	The National NeuroAIDS Tissue Consortium Brain Gene Array: Two types of HIV-associated neurocognitive impairment	White matter, basal ganglia, frontal cortex	Affymetrix Human Genome U133 Plus 2.0 Array	Phantasus
GSE35974	Liu C, Chen C, 2012	Expression data from the human cerebellum brain	Cerebellum	Affymetrix Human Gene 1.0 ST Array	Phantasus

GSE35977	Liu C, Chen C, 2012	Expression data from the human parietal cortex brain	Parietal cortex	Affymetrix Human Exon 1.0 ST Array	Phantasus
GSE46706	Ryten M et al, 2013	Expression data generated from post-mortem human brain tissue originating from neurologically and neuropathologically control individuals	White matter, medulla, cerebellar cortex, substantia nigra, thalamus, putamen, hippocampus, different cortical areas	Affymetrix Human Exon 1.0 ST Array	GEO2R
GSE44971	Lambert SR et al, 2013	Gene expression data from pilocytic astrocytoma tumour samples and normal cerebellum controls	Cerebellum	Affymetrix Human Genome U133 Plus 2.0 Array	Phantasus
GSE49036	Dijkstra AA et al, 2015	Evidence for immune response, axonal dysfunction and reduced endocytosis preceding Lewy body pathology in the substantia nigra in Parkinson's disease	Substantia nigra	Affymetrix Human Genome U133 Plus 2.0 Array	Phantasus
GSE5281	Stephan DA, Liang WS, 2006	Alzheimer's disease and the normal aged brain (steph-affy-human-433773)	Different cortical areas	Affymetrix Human Genome U133 Plus 2.0 Array	Phantasus
GSE5388	Bahn S et al, 2006	Adult postmortem brain tissue (dorsolateral prefrontal cortex) from subjects with bipolar disorder and healthy controls	Dorsolateral prefrontal cortex	Affymetrix Human Genome U133A Array	Phantasus
GSE5389	Bahn S et al, 2006	Adult postmortem brain tissue (orbitofrontal cortex) from subjects with bipolar disorder and healthy controls	Orbitofrontal cortex	Affymetrix Human Genome U133A Array	Phantasus
GSE5390	Bahn S et al, 2007	Expression profiling of human adult postmortem brain tissue from Down syndrome and healthy control subjects	Prefrontal cortex	Affymetrix Human Genome U133A Array	Phantasus
GSE54282	Riley BE et al, 2014	Systems-based analyses of brain regions functionally impacted in Parkinson's disease reveals underlying causal mechanisms	Striatum	Affymetrix Human Gene 1.0 ST Array	Phantasus
GSE54565	Sibille E, 2014	Expression data from human brain anterior cingulate cortex - including control samples and samples with major depression disorders (32 samples MD1_ACC)	Anterior cingulate cortex	Affymetrix Human Genome U133 Plus 2.0 Array	Phantasus
GSE54566	Sibille E, 2014	Expression data from human brain amygdala - including control samples and samples with major depression disorders (28 samples MD1_AMY)	Amygdala	Affymetrix Human Genome U133 Plus 2.0 Array	Phantasus
GSE54567	Sibille E, 2014	Expression data from human brain dorsolateral prefrontal cortex - including control samples and samples with major depression disorders (28 samples BA9_M)	Dorsolateral prefrontal cortex	Affymetrix Human Genome U133 Plus 2.0 Array	Phantasus

GSE54568	Sibille E, 2014	Expression data from human brain dorsolateral prefrontal cortex - including control samples and samples with major depression disorders (30 samples BA9_F)	Dorsolateral prefrontal cortex	Affymetrix Human Genome U133 Plus 2.0 Array	Phantasus
GSE54570	Sibille E, 2014	Expression data from human brain dorsolateral prefrontal cortex - including control samples and samples with major depression disorders (26 samples NY_BA9)	Dorsolateral prefrontal cortex	Affymetrix Human Genome U133 Plus 2.0 Array	Phantasus
GSE54571	Sibille E, 2014	Expression data from human brain anterior cingulate cortex - including control samples and samples with major depression disorders (26 samples BA25_F)	Anterior cingulate cortex	Affymetrix Human Genome U133 Plus 2.0 Array	Phantasus
GSE54572	Sibille E, 2014	Expression data from human brain anterior cingulate cortex - including control samples and samples with major depression disorders (24 samples BA25_M)	Anterior cingulate cortex	Affymetrix Human Genome U133 Plus 2.0 Array	Phantasus
GSE54575	Sibille E, 2014	Expression data from human brain orbital ventral prefrontal cortex - including control samples and samples with major depression disorders (24 samples NY_BA47)	Orbital ventral prefrontal cortex	Affymetrix Human Genome U133 Plus 2.0 Array	Phantasus
GSE7621	Ffrench-Mullen JM, 2007	Expression data of substantia nigra from postmortem human brain of Parkinson's disease patients (PD)	Substantia nigra	Affymetrix Human Genome U133 Plus 2.0 Array	Phantasus
GSE78246	Vawter MP, Rollins BL, 2016	Quantitative Trait Locus and Brain Expression of HLA-DPA1 And Shared Immune Alterations in Psychiatric Disorders	Anterior cingulate cortex	Affymetrix Human Exon 1.0 ST Array	Phantasus
GSE8397	Moran LB, Graeber MB, 2008	Expression profiling of the Parkinsonian Brain	Substantia nigra	Affymetrix Human Genome U133 Array	Phantasus
GSE92538	Hagenauer MH et al, 2016	Inference of cell-type composition from human brain transcriptomic datasets illuminates the effects of age, manner of death, dissection, and psychiatric diagnosis	Prefrontal cortex	Affymetrix GeneChip Human Genome HG-U133 Plus 2 Array	Phantasus

**Only brain areas included in the review listed*

Supplementary Table S2. Brain RNA expression datasets (high throughput RNA sequencing data)

GSE identifier	Authors	Title	Structure*	Analytic software
GSE110727	Zaghlool A et al, 2019	Characterization of the nuclear and cytosolic transcriptomes in human brain tissue	Frontal cortex (cytoplasmic and nuclear fractions)	edgeR
GSE136666	Xicoy H et al, 2020	Transcriptomic profiling of substantia nigra and putamen in Parkinson’s disease	Substantia nigra	GREIN
GSE138614	Elkjaer ML et al, 2020	Next-generation sequencing study in multiple sclerosis white matter brain lesions	White matter	edgeR
GSE160521	McClung C et al, 2020	Diurnal rhythms across the human dorsal and ventral striatum	Nucleus accumbens, putamen, caudate nucleus	edgeR
GSE80655	Bowling KM et al, 2017	RNA-sequencing of human post-mortem brain tissues	Prefrontal cortex, anterior cingulate cortex, nucleus accumbens	edgeR
GSE68559	Webb A et al, 2017	RNA sequencing of transcriptomes in human brain regions: protein-coding and non-coding RNAs, isoforms and alleles	Frontal cortex, insula hippocampus, amygdala, putamen, cerebellum, raphe nuclei	GREIN
GSE101521	Pantazatos SP et al, 2017	Whole-transcriptome brain expression and exon-usage profiling in major depression and suicide	Prefrontal cortex	GREIN
GSE123496	Itoh Y, Voskuhl RR, 2019	Human brain tissues from healthy controls and multiple sclerosis patients	Frontal cortex, parietal cortex, hippocampus, corpus callosum, internal capsule	GREIN
GSE53239	Akula N, McMahon FJ, 2014	RNA-sequencing of the brain transcriptome implicates dysregulation of neuroplasticity, circadian rhythms, and GTPase binding in bipolar disorder	Prefrontal cortex	GREIN
GSE53697	Scheckel C et al, 2016	Human brain samples from control and advanced Alzheimer's Diseased patients were subjected to RNAseq analysis to monitor RNA level changes during AD progression	Prefrontal cortex	GREIN
GSE64810	Labadorf A et al, 2015	mRNA-Seq Expression profiling of human post-mortem BA9 brain tissue for Huntington's Disease and neurologically normal individuals	Prefrontal cortex	GREIN
GSE68719	Dumitriu A et al, 2016	mRNA-Seq expression and MS3 proteomics profiling of human post-mortem BA9 brain tissue for Parkinson Disease and neurologically normal individuals	Prefrontal cortex	GREIN
GSE79666	Xing Y at al, 2016	Transcriptome sequencing reveals aberrant alternative splicing in Huntington's disease	Primary motor cortex	GREIN
GSE80336	Davis RL, Pacifico R, 2016	Transcriptome profiling of the human dorsal striatum in bipolar disorder	Dorsal striatum	GREIN

**Only structures included in analysis in accordance with inclusion criteria signed.*

Supplementary Figure S1

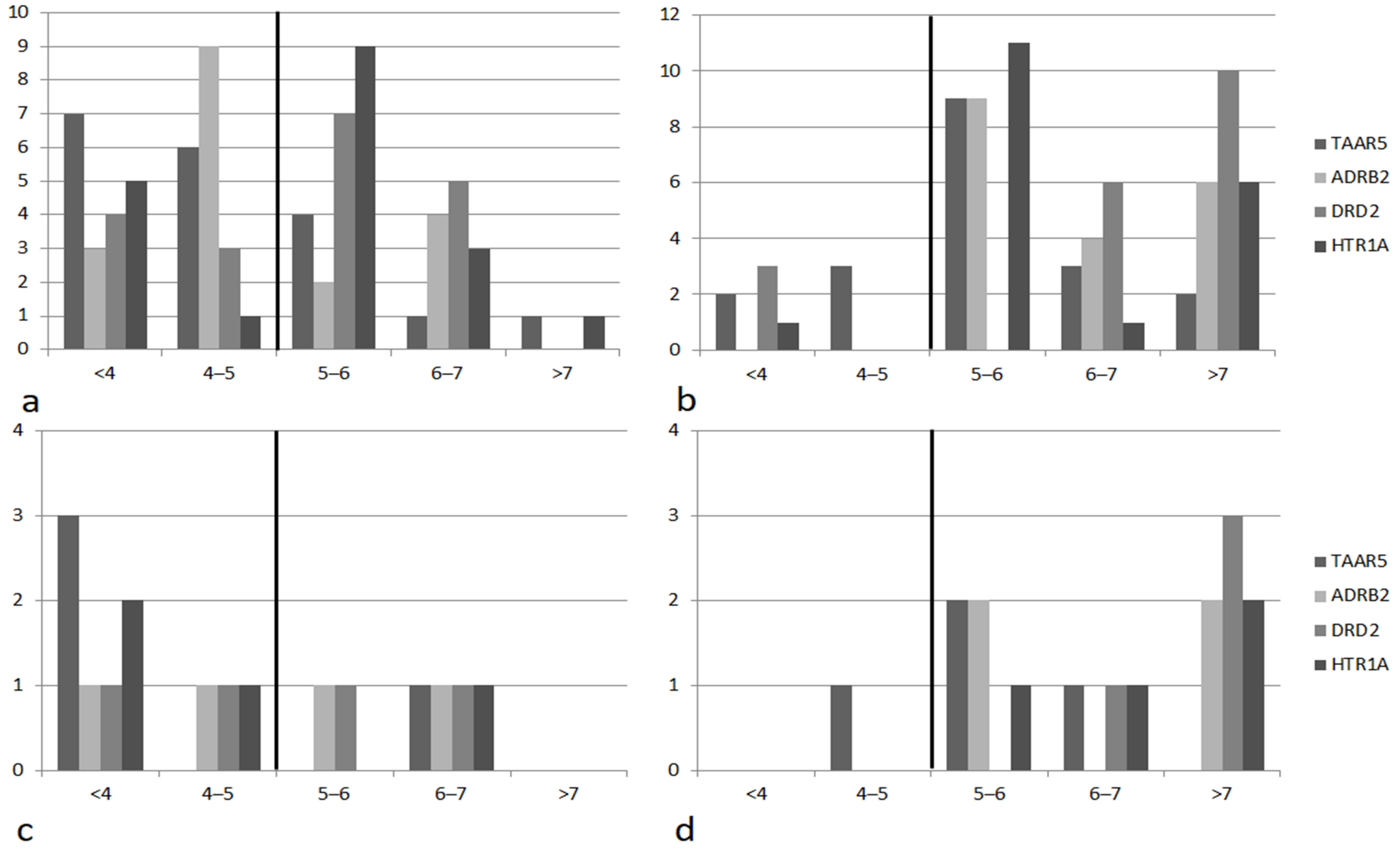

Distribution of minimal and maximal log2 normalized values for TAAR5, DRD2, HTR1A and ADRB2 in the analyzed microarray-generated datasets for neocortex and hippocampus.

a. Distribution of minimal log2 normalized values in neocortex transcriptome datasets.
b. Distribution of maximal log2 normalized values in neocortex transcriptome datasets.
c. Distribution of minimal log2 normalized values in hippocampus transcriptome datasets.
d. Distribution of maximal log2 normalized values in hippocampus transcriptome datasets.

Cut-off log2 normalized value = 5.0 signed by vertical line.

Supplementary Figure S2

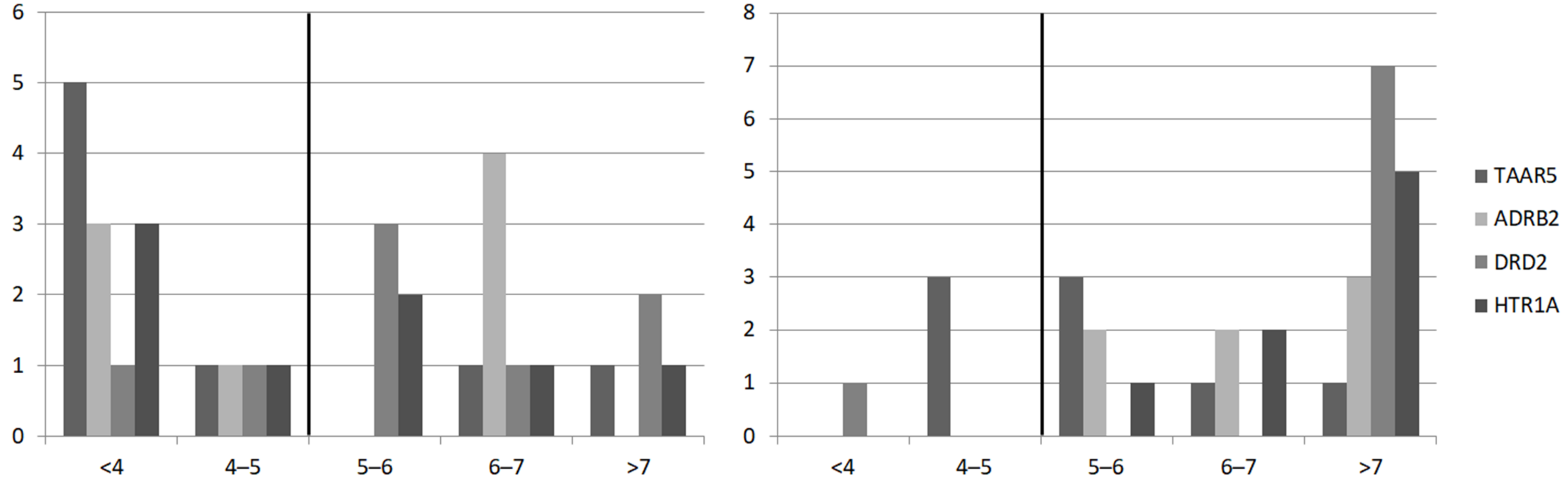

a b

Distribution of minimal and maximal log2 normalized values for TAAR5, DRD2, HTR1A and ADRB2 in the analyzed microarray-generated datasets for basal ganglia.

a. Distribution of minimal log2 normalized values in basal ganglia transcriptome datasets.

b. Distribution of maximal log2 normalized values in basal ganglia transcriptome datasets.

Cut-off log2 normalized value = 5.0 signed by vertical line.

Supplementary Figure S3

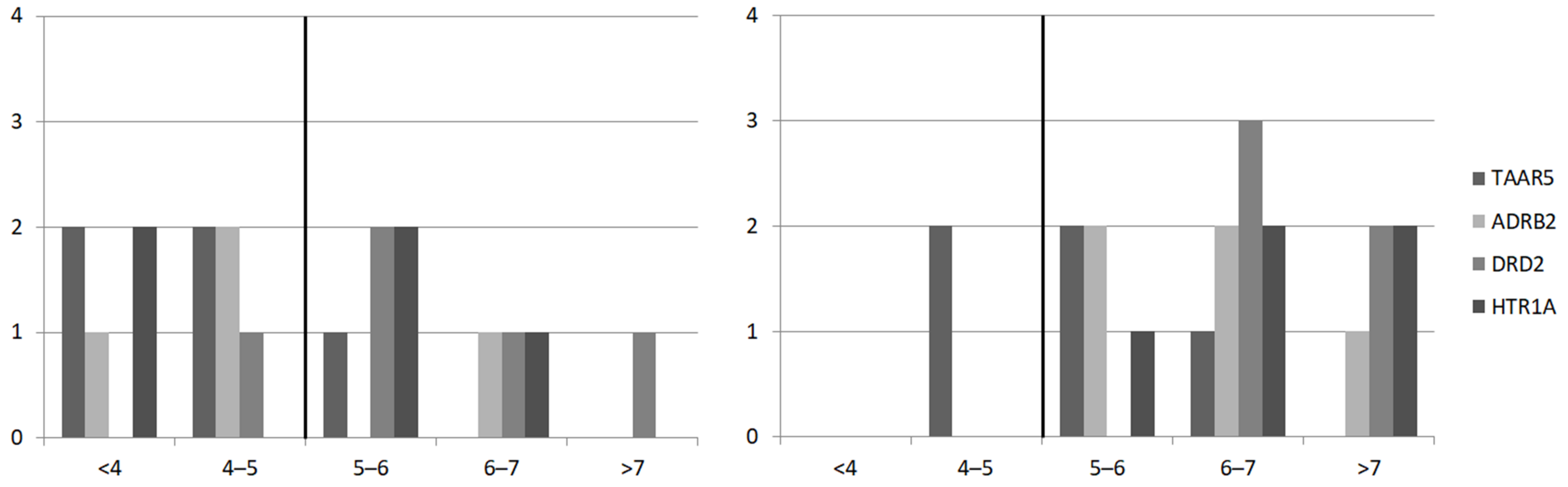

a b

Distribution of minimal and maximal log2 normalized values for TAAR5, DRD2, HTR1A and ADRB2 in the analyzed microarray-generated datasets for cerebellum.

a. Distribution of minimal log2 normalized values in substantia nigra transcriptome data sets.

b. Distribution of maximal log2 normalized values in substantia nigra transcriptome data sets.

Cut-off log2 normalized value = 5.0 signed by vertical line.

Supplementary Figure S4.

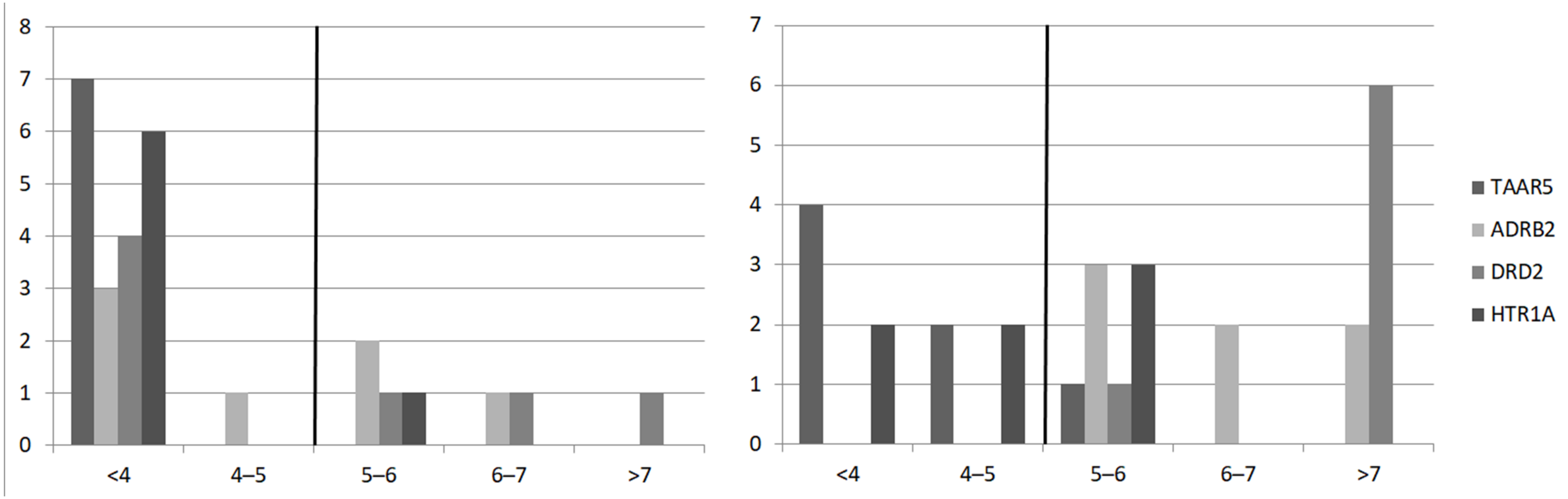

a **b**

Distribution of minimal and maximal log2 normalized values for TAAR5, DRD2, HTR1A and ADRB2 in the analyzed microarray-generated datasets for substantia nigra.

a. Distribution of minimal log2 normalized values in substantia nigra transcriptome data sets.

b. Distribution of maximal log2 normalized values in substantia nigra transcriptome data sets.

Cut-off log2 normalized value = 5.0 signed by vertical line.
